# Acetic Acid: An Underestimated Metabolite in Ethanol-Induced Changes in Regulating Cardiovascular Function

**DOI:** 10.3390/antiox13020139

**Published:** 2024-01-23

**Authors:** Andrew D. Chapp, Zhiying Shan, Qing-Hui Chen

**Affiliations:** 1 Department of Neuroscience, University of Minnesota, Minneapolis, MN 55455, USA; 2 Kinesiology and Integrative Physiology, Michigan Technological University, Houghton, MI 49931, USA; zhiyings@mtu.edu

**Keywords:** ethanol, acetic acid, acetate, nitric oxide, sympathetic nerve activity, cardiovascular function

## Abstract

Acetic acid is a bioactive short-chain fatty acid produced in large quantities from ethanol metabolism. In this review, we describe how acetic acid/acetate generates oxidative stress, alters the function of pre-sympathetic neurons, and can potentially influence cardiovascular function in both humans and rodents after ethanol consumption. Our recent findings from in vivo and in vitro studies support the notion that administration of acetic acid/acetate generates oxidative stress and increases sympathetic outflow, leading to alterations in arterial blood pressure. Real-time investigation of how ethanol and acetic acid/acetate modulate neural control of cardiovascular function can be conducted by microinjecting compounds into autonomic control centers of the brain and measuring changes in peripheral sympathetic nerve activity and blood pressure in response to these compounds.

## 1. Introduction

**Figure 1:**
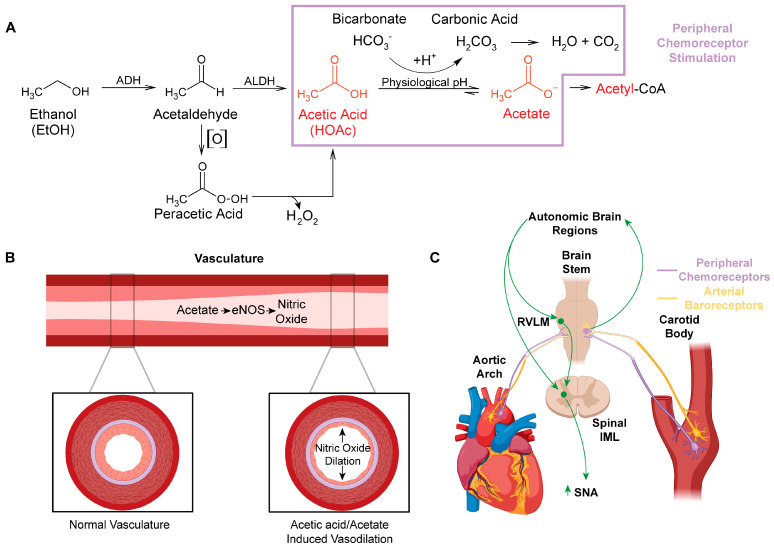
Impact of acetic acid/acetate on peripheral vasculature and the regulation of cardiovascular function. (**A**) Major metabolic pathway for ethanol metabolism (box in purple highlights potential chemoreceptor−stimulating pathways). Auto−oxidation (denoted [O]) of acetaldehyde, which can be increased by the presence of Fe^3+^ to form peracetic acid (peroxyacetic acid). Peracetic acid decomposes to acetic acid, generating hydrogen peroxide. (**B**) Acetate stimulates eNOS in the peripheral vasculature, which increases production of the powerful vasodilator, nitric oxide. (**C**) Schematic of the location of peripheral arterial baroreceptors and chemoreceptors, their innervation to the brainstem, and their relationship with neural control of autonomic function. Abbreviations: alcohol dehydrogenase (ADH), cytochrome P450 (CYP 450), aldehyde dehydrogenase (ALDH), endothelial nitric oxide synthase (eNOS), nitric oxide (NO), rostral ventrolateral medulla (RVLM), intermediolateral column (IML), sympathetic nerve activity (SNA).

Ethanol metabolism produces systemic maladaptive changes through oxidative stress. Ethanol metabolism in vivo generates reactive oxygen and nitrogen species, predominantly through oxidative pathways involving NADPH/NADP^+^, NAD+/NADH, and/or H_2_O_2_ [1]. Ethanol is oxidized to acetic acid, which becomes acetate at physiological pH (Figure 1A) [2]. Acetate is then shuttled into the citric acid cycle, where additional oxidative stress is likely generated through electron transport chain electron leak [3]. Chronic oxidative stress from ethanol metabolism leads to liver [4,5,6,7,8], brain [9,10,11,12,13], and cardiovascular pathologies [14,15,16,17,18,19,20]. 

For many years, moderate ethanol consumption was thought to provide some benefit to cardiovascular health [21,22]. This was endorsed by the American Heart Association (AHA) [23]. However, this was at odds with the literature on alcohol use disorder suggesting that chronic use of ethanol leads to the development of hypertension and cardiovascular disease [24,25,26]. By 2023, after several research articles suggested that ethanol offers no cardiovascular benefit [27,28], the AHA reversed course, warning that even one ethanol-containing drink a day can increase cardiovascular disease risk [29]. 

**Figure 2:**
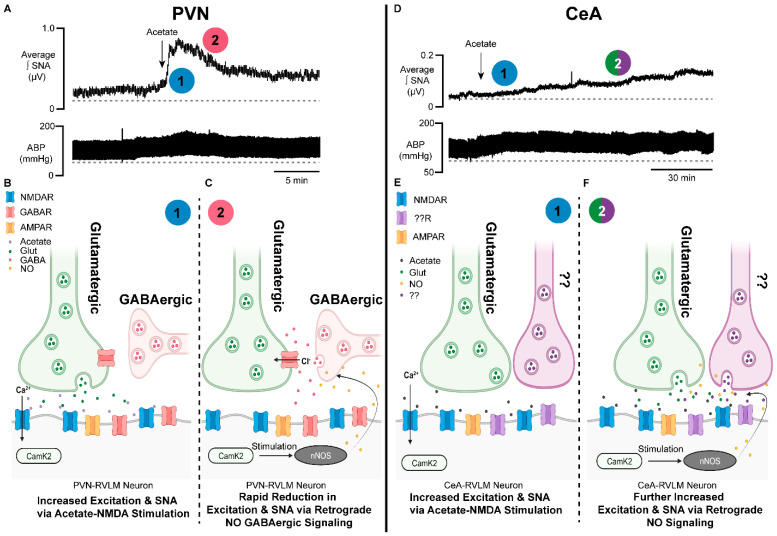
Brain-generated sympathoexcitatory responses to microinjections of acetate differ by autonomic region: implications for nitric oxide. (**A**) Representative sympathetic nerve recording and arterial blood pressure in response to PVN microinjected acetate. Note the rapid increase in sympathetic nerve activity (1, blue) and time of onset, followed by a rapid reduction in excitation (2, red). (**B**) Proposed schematic of the synaptic mechanisms contributing to the acetate-induced sympathoexcitatory response in PVN−RVLM neuronal circuitry. Note that (1, blue) corresponds to the response in (**A**). (**C**) Proposed schematic of NO retrograde synaptic signaling in PVN−RVLM neurons. Acetate activation of the NMDAR stimulates NO production, which increases presynaptic GABA release on glutamatergic synapses, reducing sympathoexcitation. Note that (2, red) corresponds to the response in (**A**). (**D**) Representative sympathetic nerve recording and arterial blood pressure in response to CeA−microinjected acetate. Note the steady increase in sympathetic nerve activity (1, blue) and the steady increase in excitation (2, green/purple). (**E**) Proposed schematic of the synaptic mechanisms contributing to the acetate-induced sympathoexcitatory response in CeA−RVLM neuronal circuitry. Note that (1, blue) corresponds to the response in (**D**). (**F**) Proposed schematic of NO retrograde synaptic signaling in CeA−RVLM neurons. Acetate activation of the NMDAR stimulates NO production, which increases the release of presynaptic glutamate and other neurotransmitters, increasing sympathoexcitation. Alternative inputs may also be propagating increased SNA. Note that (2, green/purple) corresponds to the response in (**D**). Abbreviations: paraventricular nucleus of the hypothalamus (PVN), rostral ventrolateral medulla (RVLM), neuronal nitric oxide synthase (nNOS), calcium calmodulin kinase 2 (CAMK2), nitric oxide (NO), calcium (Ca^2+^), chloride (Cl^−^), N-methyl-D-aspartate receptor (NMDAR), gamma aminobutyric acid receptor (GABAR), sympathetic nerve activity (SNA), glutamate (Glut), gamma aminobutyric acid (GABA), α-amino-3-hydroxy-5-methyl-4-isoxazolepropionic acid receptor (AMPAR).

A major gap in the ethanol research field is the limited investigation of the role of acetic acid/acetate in the pathologies associated with ethanol use. Acetic acid/acetate has been assumed to be a relatively benign compound [1], as acetate is a feedstock for the generation of ATP through the citric acid cycle [30] and for the generation of acetyl-CoA and acetylation reactions [31,32]. However, acetic acid is linked to several distinct disease states, including Alzheimer’s disease [33,34], neurodegenerative disease [33,35], obesity [36], and gut–brain dysbiosis [33,35,36]. In this review article, we highlight how acetic acid/acetate may influence the regulation of cardiovascular function through several mechanisms, including the generation of reactive oxygen and nitrogen species (Figure 1 and Figure 2).

## 2. Acetaldehyde and Peracetic Acid: All Roads Lead to Acetic Acid/Acetate

Acetaldehyde has been one of the most highly researched ethanol metabolites and has been implicated in the generation of reactive oxygen species, protein adduct formation [37,38,39], the increased production of salsolinol [40,41,42,43,44,45,46], and neuronal toxicity [47,48,49,50]. In individuals with aldehyde dehydrogenase (ALDH) 2 deficiencies, the buildup of acetaldehyde is thought to cause flushing, nausea, and headache [51,52,53,54]. Disulfiram, an ALDH inhibitor and alcohol use deterrent, also increases the buildup of acetaldehyde, which is thought to be the mechanism of action for reducing alcohol consumption [55,56,57]. 

Interestingly, blood acetaldehyde concentrations are not easily measured and have been reported in the range of 10–40 nM when blood ethanol concentrations are 10–20 mM [58,59]. Part of the prevailing theory on why blood acetaldehyde concentrations are so low is due to its rapid metabolism to acetic acid [1]. And while this may be true, there are other chemical factors and conversion routes for acetaldehyde that may be contributing to its low detection. 

First, acetaldehyde is a volatile liquid that is soluble in aqueous solutions [60]. Its boiling point is 20.2 °C (68 °F) [60], much lower than the physiological temperature of 37 °C (98.6 °F). Thus, it is highly likely that any acetaldehyde that is not converted to acetic acid/acetate and remains in circulation passing to the lungs is exhaled in its gaseous state. Second, in a report published in 1929 by Bowen and Tietz, they noted that acetaldehyde shaken in the presence of oxygen (auto-oxidation) produced a peroxide, identified as peracetic acid [61]. These authors also noted that this type of reaction and similar reactions produced a “long chain mechanism” [61], which would be consistent with what is now a free radical-type mechanism. Indeed, follow-up work in 1950 by Bawn and Williamson further elucidated that aqueous solutions of acetaldehyde were also capable of auto-oxidation, producing the peracetic acid reported by Bowen and Tietz [61], and that these reactions could be increased and/or induced in the presence of metals, of note, iron III (Fe^3+^) [62]. Interestingly, hemoglobin, the iron-containing protein that is responsible for oxygen and carbon dioxide transport in the blood, has an iron oxidation state of Fe^3+^ when oxygen is bound [63]. This close proximity of oxygen and Fe^3+^ with any circulating acetaldehyde may convert acetaldehyde into peracetic acid, and account for the low to undetectable circulating levels following ethanol consumption [59]. 

Peracetic acid (peroxyacetic acid) is a peroxide of acetic acid. It is utilized as a broad-spectrum disinfectant, displaying bactericidal, virucidal, fungicidal, and sporicidal properties [64]. In cell toxicity studies, peracetic acid was more cytotoxic than sodium hypochlorite (bleach) [65]. Moreover, given that peracetic acid is an organic peroxide, it undergoes similar homolysis reactions as other peroxides do [66]. As such, the spontaneous or metal-catalyzed homolysis reactions of peroxides generate free radicals [66,67] and these free radicals are thought to induce cellular and tissue damage [68,69]. Hydrogen peroxide is a classic example of a peroxide used as a source of oxygen-derived free radicals in research studies [70]. Additionally, hydrogen peroxide also interacts with iron in the well-known Fenton reaction to generate additional sources of free radicals that can lead to tissue damage [71]. Similar to the Fenton reactions involving hydrogen peroxide, peracetic acid was also found to undergo such Fenton reactions with Fe^3+^, generating peroxyl (CH_3_C(O)OO•), alkoxyl (CH_3_C(O)•), and hydroperoxyl (HO_2_•) radicals [72]. In light of this information, ethanol and alcohol use disorder (AUD) researchers should consider the potential that many of the deleterious effects attributed from acetaldehyde might be due to its downstream metabolite, peracetic acid (Figure 1A).

## 3. Active Metabolites of Ethanol

Among the plethora of research on ethanol and AUD, few studies have progressed past acetaldehyde in the ethanol metabolic pathway. This is not to say that ethanol and AUD research has not entertained the possibility that acetic acid/acetate may be involved in the effects of ethanol consumption. Rather, it was more likely assumed that since acetic acid/acetate directly fed into the citric acid cycle to generate ATP, it had to be rather benign [1]. Indeed, glucose and L-lactate are both feedstock for the generation of ATP [73] and at face value, would appear benign. Glucose is consumed and L-lactic acid/lactate can be released into circulation and rapidly cleared following exercise [74]. However, both are kept within tight tolerance in humans and drastic deviations from these homeostatic values (glucose 4.0–6.0 mM [75] and L-lactic acid/lactate <2.0 mM) [76] can produce profound effects on physiological function [75,76]. As an example, an individual with diabetes whose blood glucose levels drop too low can display symptoms including confusion, shaking, nausea, vision changes, loss of consciousness, seizure, and/or death [75]. Thus, as there are tight homeostatic tolerances for glucose, the same may perhaps apply to acetic acid/acetate. 

To the best of our knowledge there is no “normal” definition of blood acetate levels in humans. Blood acetate concentrations reported in humans and rodents have been between 0.05 and 0.6 mM [77,78,79,80,81,82], and in individuals with AUD, 0.9 mM [82]. However, a majority of these studies utilized older techniques for quantifying acetate, such as volatile derivatization reactions followed by organic extractions and gas chromatography [81], which may underestimate the actual concentrations (see Chapp et al. [83]). We have recently adapted ion chromatography methodology to measure short-chain fatty acids including acetic acid/acetate in rodents, with potential applicability to humans [83]. 

In rats, we have measured baseline serum acetate concentrations of 0.23 ± 0.04 mM [83]. In rats administered a dose of ethanol (2 g/kg), equivalent to a blood alcohol concentration (BAC) of 0.2% the time of metabolism influenced serum acetate concentrations, with peak serum acetate concentrations measured at ~4.2 mM in male rats and ~3.9 mM in female rats (unpublished data). In C57BL/6J mice, the baseline serum acetate level in males was 0.63 ± 0.04 mM and in females was 0.56 ± 0.6 mM [2]. Similar to the rats, mice administered ethanol (2 g/kg) had serum acetate concentrations influenced by metabolism times, with peak values of 3.67 mM in males and 3.60 mM in females [2]. These data demonstrate that: (1) Baseline serum acetate levels can potentially differ between rats and mice (~0.1–0.2 mM in rats and ~0.5–0.6 mM in mice) and thus may also be different in humans if measured using the same technique [83]. (2) The same dose of ethanol seems to produce similar serum acetate concentrations, at least between rats and mice. Again, whether this highly intoxicating dose of ethanol (2 g/kg, BAC ~0.2%) or lower doses (1 g/kg) in humans produces the same serum acetate concentrations as it does in rodents remains to be determined. We have, however, shown that these concentrations of acetate in dopaminergic-like PC12 cells can increase cytosolic calcium in an NMDAR-dependent manner, as well as increase cytosolic reactive oxygen species [84].

## 4. Peripheral Actions of Acetic Acid/Acetate: Impact on Nitric Oxide Synthase (NOS) and Nitric Oxide (NO)

Ethanol in humans has been found to influence heart rate, cardiac output, stroke volume, and sympathetic nerve activity [85,86,87,88,89,90,91]. It causes both vasoconstriction and vasodilation [14,92,93]. In rodent models, ethanol has been shown to increase the bioavailability of nitric oxide [93,94,95]. However, these studies did not account for ethanol metabolism or the production of acetic acid/acetate. 

In studies investigating the effect of buffering agents in hemodialysis solutions, it is observed that acetate-based solutions create greater cardiovascular and hemodynamic instability than bicarbonate-based solutions do [96,97]. These instabilities include vasodilation, hypoxemia, increased cardiac output, and angina pectoris [98,99,100]. Methodical investigation identified that these acetate-based hemodialysis solutions increased levels of nitric oxide synthase (NOS) and nitric oxide (NO) [101,102], a powerful radical which stimulates vasodilation [103,104,105]. Several studies have also identified acetate-induced elevations in tumor necrosis factor alpha (TNFa) and cyclic adenosine monophosphate (cAMP), both mediators of NOS [102]. This was seen in dialysis buffers containing as little as 4 mM acetate [102]. A likely mechanism through which ethanol produces vasodilation is through ethanol’s metabolism to acetic acid/acetate, which stimulates production of NOS and NO (Figure 1B). This finding was also substantiated by Sakakibara S et al., who found that consumption of vinegar increased eNOS production with as little as 200 uM acetate in both early-phase detection (20 min) as well as late-phase detection (4 h) [106]. Furthermore, they found increased forearm vasodilation in adults who had consumed acetic acid compared to a control. This time course of action for acetic acid/acetate suggests a profound impact on peripheral eNOS even with minimal concentrations of and exposure to acetate [106]. 

## 5. Direct Effects of Ethanol and Acetic Acid/Acetate on Cardiac Function

The potential of ethanol consumption to influence cardiac function has been well documented. In the acute setting, ethanol can increase cardiac output [107] and reduce plasma potassium [108], which may contribute to ethanol-induced arrhythmia or “holiday heart” [109]. The acute effects of ethanol on contractility appear to be mixed, with some studies suggesting either no change [107,110,111] or a decrease [112,113] in cardiac contractility. Chronic ethanol use also contributes to cardiomyopathy [114,115] and arrhythmias [20]. 

Similar to the effects of acute ethanol, acute acetate is also capable of altering cardiac function. Acute acetate administration has been found to increase cardiac output [116,117], precipitate hemodynamic instability [97,101,116], and reduce myocardial contractility [118,119]. Interestingly, some of the direct cardiac effects of acetate were speculated to be tied to acetate-induced NO release [101]. Indeed, the direct cardiac effects of NO are bimodal, with lower concentrations of NO increasing the ionotropic effects [120] and high concentrations of NO reducing cardiac myocyte contractility [121]. Whether the mixed effects of ethanol on cardiac function are a result of acetate and/or acetate-induced NO release remains to be determined. Furthermore, the effect of chronically elevated acetate from chronic ethanol use and metabolism on cardiac function has yet to be investigated. It is provocative to postulate that the major compound driving alcohol-induced cardiomyopathy [122,123] may be acetate rather than ethanol, and as such, future studies of chronic acetate seem warranted.

## 6. Ethanol Metabolism to Acetic Acid Alters Acid/Base Homeostasis and Likely Engages Peripheral Chemoreceptors

Although ethanol itself is not acidic (pKa value ~16) [124], ethanol acts as an exogenous source of acidic hydrogens. Once ethanol is fully oxidized to acetic acid (pKa value ~4.78) [125], the labile acidic hydrogen becomes >95% dissociated at physiological pH [126]. pKa is a measure of hydrogen acidity, as a review of pKa (see *General Chemistry*, sixth edition, Julia Burdge). The higher the pKa value, the less acidic the hydrogen. At physiological pH, acetic acid likely reacts with the bicarbonate buffering system (i.e., the vinegar and baking soda reaction), forming acetate and carbonic acid (Figure 1A). Carbonic acid is then broken down into water and carbon dioxide. The acidic hydrogen on acetic acid and the excess carbon dioxide cause activation of (1) peripheral chemoreceptors located in the carotid sinus and aortic arches, and (2) neural control centers in the brain, which control sympathetic outflow and increase sympathetic nerve activity (SNA) (Figure 1C).

In the United States, humans are considered legally intoxicated at a blood alcohol concentration (BAC) of 0.08% or a blood serum concentration of ~17 mM ethanol [127]. The conversion of ethanol to acetic acid is nearly 1:1, with ~95% of ethanol converted to acetic acid [128]. Thus, for 17 mM of ethanol consumed, the predicted amount of acetic acid produced would be ~16–17 mM. To buffer or neutralize this concentration of protons, roughly 17 mM of bicarbonate would be consumed. The normal concentration of bicarbonate in the serum is ~23–28 mM [129,130]. This bicarbonate consumption creates a high acid load, generating compensatory mechanisms within the kidneys and lungs to maintain pH homeostasis [130,131,132]. 

## 7. Integration of Arterial Baroreceptors, Chemoreceptors, and NO Signaling in Response to Acetic Acid Generated by Ethanol Metabolism

The integration of the peripheral cardiovascular regulatory systems engaged by acetic acid likely explains the complex phenomenon of mixed vasodilation and vasoconstriction induced by ethanol. First, acetic acid increases NOS activity and NO production, which leads to vasodilation [98,101,102]. Arterial baroreceptors located in the carotid sinus and aortic arch [133] would relay the resultant drop in blood pressure to neural control centers, which regulate cardiovascular function [134] (Figure 1C). These neural control centers then increase SNA to constrict the vasculature in an attempt to maintain homeostasis [133]. Third, the acid load generated from the acetic acid and/or excess carbon dioxide would similarly be sensed by the peripheral chemoreceptors, also located in the carotid sinus and aortic arch. And, in the same type of neural feedback loop as the arterial baroreceptor activation, chemoreceptor activation would also increase SNA and constrict the peripheral vasculature [135,136,137,138]. Thus, the complex vasoconstriction and vasodilation effects of ethanol consumption [93] can be explained when looking at the effects being driven by acetic acid and not ethanol per se. 

## 8. Acetic Acid-Induced Changes in Neural Control of Cardiovascular Regulation

Acute oral consumption of ethanol consistently increases arterial blood pressure (ABP) and SNA in humans [85,89,90,91] and rodents [139,140,141]. Likewise, chronic ethanol use has been shown to increase norepinephrine, SNA, and ABP [111,142,143,144,145,146]. In one acute human study, dexamethasone was found to blunt ethanol’s sympathoexcitatory effect [90] through a reduction in corticotropin-releasing hormones (CRHs), suggesting that this response is at least partially mediated by central effects. In rodent studies, oral ethanol has been less consistent [92], with some studies showing an increase in SNA and ABP [143,147] and other studies showing decreases in ABP [108,110]. As some other reviews have noted, the timing of measurements in rodents may be important [24]. In one rodent study, Crandall et al. observed that ABP was normal at peak blood ethanol concentrations but was significantly elevated 24 h post ethanol dosing [148]. Interestingly, we now know from pharmacokinetic studies that this time point corresponds to when serum acetate is still elevated, typically remaining elevated for 12–24 h post ethanol metabolism [149,150]. As such, reasonable evidence exists that suggests acetate may be a lead compound in driving the ethanol-induced effects on cardiovascular function from a centrally mediated standpoint.

When attempting to elucidate the neuronal mechanisms of ethanol-induced changes in cardiovascular regulation, several brain regions that regulate autonomic function have been studied. The rostral ventrolateral medulla (RVLM) is a significant brain region involved in the integration of upstream brain regional sympathoexcitatory outputs [139,151,152,153,154,155,156,157,158]. The RVLM projects to the spinal intermediolateral column (IML), which is the first synapse in the central to peripheral sympathetic output pathway [158,159]. In direct application studies of the RVLM conducted by El-Mas and colleagues, ethanol microinjection dose-dependently increased RVLM norepinephrine and ABP in spontaneously hypertensive rats (SHRs) [141,160]. Additional follow-up studies by the same group suggested that this ethanol response was due to enhanced catabolism of ethanol to acetaldehyde [141]. They however indirectly speculated that acetate had little effect on increasing ABP levels [141]. Interestingly, when investigating catalase and ALDH enzyme activity in the RVLM between SHRs and non-hypertensive control (Wistar–Kyoto, WKY) rats, the SHRs had higher levels of catalase activity compared to the WKY rats, with no differences in ALDH activity, suggesting a potential genetic component in relation to differences in brain ethanol metabolism [141]. While this study described above did not detect differences in ALDH activity, a recent and exciting report has highlighted brain region differences in ALDH2 expression in mice [161]. This previous finding suggests that investigators must be cognizant of how brain regions and/or genetic differences in ethanol metabolism may influence behavior and/or cardiovascular function. 

The RVLM integrates projections from the paraventricular nucleus (PVN-RVLM) and the central nucleus of the amygdala (CeA-RVLM) [139,151,152,153,154,155,156,157,158], two brain regions that regulate autonomic function [139,151,152,153,154,162]. We have previously shown that microinjection of 1.7 µmole ethanol into the CeA consistently increases SNA and ABP [139]. This ethanol-induced pressor response within the CeA was *N*-methyl-D-aspartate receptor (NMDAR)-dependent [139,151], countering the predominant theory in the alcohol research field of ethanol inhibiting NMDAR function [163]. Furthermore, inhibiting glutamatergic receptors in the RVLM blunted the effect of ethanol microinjected in the CeA, suggesting that the sympathoexcitatory response acted through CeA-RVLM circuitry [139]. Moreover, we speculated that the CeA–ethanol response may have been at least partially driven by local brain metabolism of ethanol to acetate. We microinjected 0.2 µmole sodium acetate in the CeA and were able to elicit responses in the SNA similar to those achieved with ethanol. This acetate-induced response was also blunted by the NMDAR antagonist, AP5 [139]. Our follow-up whole-cell electrophysiology studies in brain slices containing CeA-RVLM-projecting neurons demonstrated that (1) acetate increased CeA-RVLM neuronal excitability in a dose-dependent manner, (2) the excitation was driven by acetate and not sodium concentrations, (3) the acetate-induced increase in CeA-RVLM neuronal excitability was sensitive to NMDAR antagonists, AP5 and memantine, and (4) acetate directly stimulated NMDAR-sensitive currents and cytosolic calcium increases, which were also abolished by NMDAR antagonists [151]. 

While we have shown that acetate appears to activate NMDAR in the CeA of rats, this finding has also been replicated in C57BL/6J mice in the nucleus accumbens shell [2,164], a key node in the mammalian reward circuit. Surprisingly, the effect of acetic acid-induced activation of the NMDAR was greater in females compared to males, highlighting a potential mechanism contributing to females’ accelerated development of alcohol use disorder and the greater neuronal degeneration associated with long-term use in females when compared to males [2]. 

## 9. NMDAR–NO Interactions

NO activation and NMDAR activation are intricately tied together [165]. An increase in brain NO has been shown to lead to NMDAR activation [166], and oppositely, NMDAR activation is capable of stimulating NO production [165,166,167]. NO, which is a soluble and diffusible gas [168], can then enhance presynaptic neurotransmitter release, such as the release of glutamate, and further potentiate an excitatory response through retrograde signaling [169,170]. In the central nervous system, neuronal nitric oxide synthase (nNOS), inducible nitric oxide synthase (iNOS), and endothelial nitric oxide synthase (eNOS) are all present [171]. In neurons, nNOS–NMDAR interactions are governed predominantly by cytosolic calcium and calcium calmodulin kinase II (CamK2) [172]. Increased NMDAR activation stimulates CamK2, resulting in increased nNOS, which stimulates the production of NO and cyclic guanosine monophosphate (cGMP), which then diffuses out into the synaptic cleft where it can modulate neuronal function [173]. 

In the RVLM, NO was found to be excitatory, and this was likely through NO-dependent glutamatergic activation [174,175,176]. In our studies investigating the impact of acetate on NMDAR and neuronal function, we had hypothesized that acetic acid/acetate was capable of mimicking the structures of glutamate and glycine [139,151], accounting for the direct effects seen at the NMDAR during in vivo and ex vivo experiments. However, it is possible that acetic acid/acetate may also be stimulating NMDAR and glutamatergic activation [169,170] via increased NO, which then may stimulate presynaptic glutamate release and/or postsynaptic NMDAR activity.

Thus, in addition to the effect of acetate generation of NO on the peripheral vasculature, there is the potential for acetate to stimulate NO production in the brain. NO has been shown to have direct neuromodulatory effects on several neurotransmitters and signaling pathways in the brain, and this includes NMDA [166,167,170,171], GABA [177,178,179,180], and glutamatergic signaling [174,175,176]. These ligand-gated ion channels and neurotransmitters are impacted in ethanol studies [181] and as such, the possibility of acetic acid/acetate-generated NO production may be a major driving force in altered neuronal function post ethanol exposure.

The existence of temporal sympathoexcitatory effects between PVN and CeA microinjections of acetate also lends credibility to an acetate–NMDAR–NO interaction (Figure 2). The PVN has been extensively studied in relation to neural control of cardiovascular function [145,152,153,154,155,156,157,162,177,178,179,180,182,183,184,185,186,187,188], while for the CeA, such research is much less common [139,151,189]. The PVN contains an abundance of GABAergic presynaptic terminals [155,190,191] and these terminals have been shown to be modulated by NO [178,179,180,192,193], which can influence cardiovascular function. In an elegant study from Li, Mayhan and Patel, this group found that microinjection of NMDA into the PVN elicited increases in SNA, ABP, and HR [192]. To investigate the contribution of NO in this response, the group included a nitric oxide synthase inhibitor, N(G)-monomethyl-L-arginine (L-NMMA), with and without NMDA. PVN microinjections of L-NMMA and NMDA produced significantly larger pressor responses compared to NMDA alone [192]. Thus, the inhibition of NO production with L-NMMA was found to exacerbate NMDAR sympathoexcitation in the PVN. To put this another way, PVN NMDAR activation generated NO, which acted as an inhibitory break on sympathoexcitation. Finally, the group found that administration of the NMDAR antagonist AP5 blunted these effects, demonstrating that they were NMDAR-dependent. This same group (Li and Patel et al.) identified that the NMDAR–NO inhibitory break within the PVN was mediated by NMDAR activation, leading to NO modulation of the GABA release on presynaptic glutamate [192]. 

Sympathoexcitatory responses to the PVN microinjection of acetate, which are sensitive to NMDAR antagonists, display a quick on/off response relative to CeA-microinjected acetate (Figure 2A). As has been previously discussed above, the PVN contains strong GABAergic inputs on glutamatergic synapses, which have been demonstrated to be inhibited by a retrograde NMDAR–NO signaling mechanism [192]. We hypothesize that the quick excitatory acetate response to PVN microinjection (Figure 2A,B) followed by a rapid normalization to baseline (Figure 2A,C) is likely due to an acetate–NMDAR–NO–GABA release type of mechanism. This PVN response can be contrasted to those we have observed from CeA-microinjected acetate (Figure 2D).

Contrary to the abundance of GABAergic inputs on presynaptic glutamatergic inputs to the PVN, presynaptic inputs to the CeA appear as a heterogenous mixture of glutamatergic [194], GABAergic [195], serotonergic [196], and norepinephrinergic [197] projections. However, a recent review has also suggested the potential for heavy glutamatergic innervation to the CeA [198]. We have demonstrated in this brain region an acetate–NMDAR effect both in vivo [139] and also ex vivo [151]. Given the blend of presynaptic projections to the CeA and the limited to non-existent mapping on CeA-RVLM neurons, which we have studied the effects of acetate on [139,151], it is unclear as to what, if anything, may be presynaptically influencing this autonomic circuitry. Similar to the NMDAR–NO effect within the PVN [192], a potential NMDAR–NO effect in the CeA is also a strong possibility (Figure 2D–F). However, given the slower response time and maximum sustained pressor responses to CeA-microinjected acetate relative to the PVN (Figure 2A,D), we speculate a potential acetate–NMDAR–NO retrograde signaling response that is more aligned with a mixed-mechanism involving glutamatergic and G-protein-coupled receptor responses (Figure 2E,F). 

## 10. Intersection of Alcohol Cardiovascular Research and AUD Research

While alcohol consumption and its associated cardiovascular sequelae are a component of AUD pathophysiology, continued alcohol use despite negative outcomes remains the major issue driving secondary pathologies [199,200,201]. Thus, understanding the key neurobiological mechanisms which contribute to the rewarding aspects of alcohol use and those which eventually lead to the development of AUD is crucial for the development of treatment options for individuals suffering from AUD. The investigation of how alcohol metabolism (the generation of acetic acid/acetate) influences neural control of cardiovascular function intersects directly with researchers investigating the neurobiology of AUD, as the key mechanisms driving both are likely translatable across brain regions. We have highlighted the effect of acetic acid/acetate and ethanol on different organs/organ systems in Table 1. 

Indeed, we have directly tested this hypothesis and have shown that acetic acid at physiologically relevant concentrations applied to medium spiny neurons (MSNs) of the nucleus accumbens shell (NAcSh), a key node in the mammalian reward circuit, is capable of increasing presynaptic glutamate release and increasing neuronal excitability [164]. In a subsequent follow-up study, we identified sex differences in acetic acid-induced NMDAR responses (females > males) in this same brain region [2]. Whether the acetic acid-induced increase in presynaptic glutamate release is mediated by presynaptic acidification [202,203], presynaptic NMDAR activation, retrograde NMDAR–NO signaling, and/or a combination of all of these remains to be determined. However, the stark sex difference in acetic acid-induced NMDAR responses may potentially underlie the greater propensity for females to experience accelerated AUD and suffer greater alcohol-induced neurodegeneration compared to males.

**Table 1:** Impact of ethanol and acetic acid/acetate on different organs/organ systems.

Organ/Organ System	Acetic Acid/Acetate	Ethanol
Brain	↑GABA [161,204], ↑Glutamate [164,204], ↑NMDAR [2,84,139,151], ↑Dopamine [203], ↑Neuropathology [34,35,84], ↑Cerebral blood flow [205]	↑GABA [195,206], ↓Glutamate [207,208], ↑Glutamate [206,209], ↑Dopamine [209,210,211], ↑Serotonin [211,212] ↓NMDAR [163,213], ↑Neuropathology [13,214,215], ↑Cerebral blood flow [216,217]
Heart	↓Contractility [119,218], ↑O_2_ consumption [116], ↑Coronary flow [116], ↑Cardiac output [116]	↓Contractility [92,219], ↑Coronary flow [220], Arrhythmia [221], ↑Cardiac output [107]
Gastrointestinal	↑Inflammation: Oral cavity [222,223], Esophagus [224], Stomach [225], Small intestine [226], Liver [8], Colon [227]	↑Inflammation: Oral cavity [228], Esophagus [228], Stomach [229], Small intestine [230,231], Liver [8,232,233], Colon [230]
Vasculature	↑Vasodilation [98,99,102,106,119], ↑NOS [102,106], ↑NO [102]	↑Vasodilation [85,93,146,234], ↑NOS [235,236], ↑NO [93,95,236]

Note: This table is not an exhaustive list, ↑ = increase, ↓ = decrease.

## 11. Conclusions and Future Research Directions

Given the complex effects of alcohol on cardiovascular function and neural control of the autonomic nervous system, future studies are needed to determine the mechanisms of these effects. For instance, there is a substantial body of research in the literature on the impacts of alcohol consumption on the peripheral vasculature [93,234]. However, many of these studies have not explored how alcohol-induced changes in the peripheral vasculature can affect neural control of the sympathetic nervous system (e.g., alcohol peripheral vasculature dilation stimulating arterial baroreceptors and initiating neural sympathoexcitation to maintain homeostasis). This push–pull dynamic between the peripheral and central nervous systems may be a contributing factor to the development of alcohol-induced hypertension [15,24,25,26,90,142,145,199] and cardiomyopathy [122,123]. While one could make an argument that moderate ethanol consumption provides cardiovascular benefits (e.g., increased vasodilation and cardiac output), these effects are likely countered by the acetic acid/acetate-initiated peripheral chemoreceptor reflex, arterial baroreceptor reflex, and activation of pre-sympathetic neurons in the central nervous system, which would increase SNA. Chronic ethanol use, and elevated acetic acid/acetate from its metabolism, would be anticipated to contribute to end organ damage due to increased sympathetic outflow and is potentially further exacerbated by ethanol/acetate-induced dysregulation of NO signaling. Dysregulation of NO signaling is a major finding in essential hypertension [237,238,239,240]. Furthermore, since alcohol and acetic acid/acetate are capable of crossing the blood–brain barrier, the impact of the brain’s metabolism of ethanol on neural control of cardiovascular function also needs to be studied. 

Future studies using rodent models could investigate the following: (1) What is the net output of ethanol and/or its metabolism in the brain on SNA and ABP? (2) Are responses to locally microinjected acetate in the PVN and CeA blunted or exacerbated by manipulating NO signaling? (3) How might acetic acid/acetate-induced NO signaling in the brain influence AUD and alcohol-related neurodegeneration? 

The neural signaling mechanisms influencing cardiovascular function listed above share potential overlapping mechanisms with the development and maintenance of AUD. Thus, in vivo microinjection studies offer a powerful pharmacological tool to evaluate the excitatory/inhibitory actions of ethanol and its metabolites in real time by measuring sympathetic nerve activity and arterial blood pressure. Future studies and findings within this area of research may be beneficial for the development of treatment strategies for not only alcohol-induced cardiovascular diseases, but also for AUD.
